# Stress Fracture of the Hamate Hook in a Water Polo Player

**DOI:** 10.1155/2019/2483142

**Published:** 2019-03-13

**Authors:** Hana Ueda, Shunpei Hama, Masataka Yasuda, Kenta Minato, Masahiro Miyashita, Kishik Shin

**Affiliations:** Department of Orthopaedic Surgery, Baba Memorial Hospital, 4-244 Nishiku Hamadera Funao-cho Higashi, Sakai, Osaka 592-8555, Japan

## Abstract

Hamate hook fractures are usually caused by direct trauma while using a tennis racket or a baseball bat. We report stress fracture of the hamate hook in a water polo player without any specific trauma. We consider that the stress fracture occurred via indirect mechanisms through the flexor tendons. Strong ulnar deviation of the wrist during ball release and strong grip on a ball with outstretched fingers, which are unique to water polo, were the likely causes of the stress fracture of the hamate hook.

## 1. Introduction

The hamate hook is an eminence of bone at the hypothenar region and the attachment point for the flexor digiti minimi brevis, abductor digiti minimi, flexor retinaculum, and pisohamate ligament as well as a pulley for the flexor tendons. Fractures of the hamate hook are usually caused by direct trauma while playing racket sports, baseball, or golf [1]. We report stress fracture of the hamate hook in a 17-year-old water polo player without any direct trauma, likely caused by indirect mechanisms.

## 2. Case Presentation

A 17-year-old male water polo player presented with pain in the ulnar side of the right wrist for the past 3 months. He is a member of the water polo team at his high school, and his level was high enough to participate in the interscholastic water polo competition. After shooting a goal 1 week ago, his pain increased; however, there was no previous direct trauma to the right wrist. Upon an initial presentation to a private clinic, no specific injury was diagnosed, and he was then referred to our institution.

**Figure 1:**
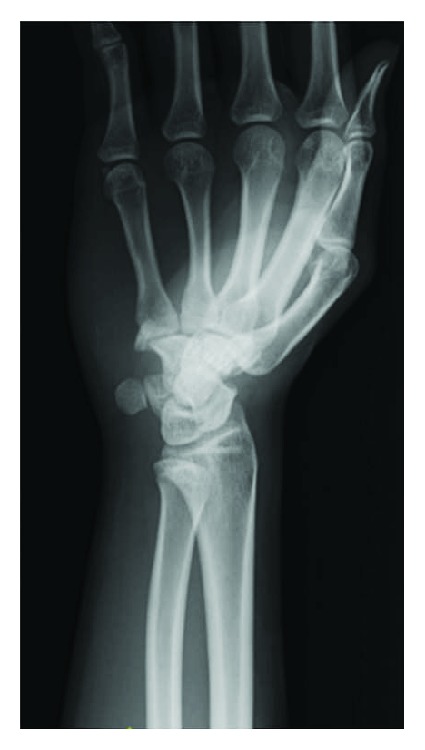
Radiograph taken at the previous clinic in supinated oblique position.

Upon initial physical examination, he had tenderness on the hypothenar region and ulnar pain when moving the wrist. The grip strength of his right hand was 10 kg, contrasted with 44 kg of the left hand. Radiographs taken in the previous clinic did not show any injury (Figure 1). Tenderness around the pisiform was also noted, and because arthritis of the joint was suspected, triamcinolone acetonide was injected into the pisotriquetral joint region.

One week after the injection, he showed improved tenderness around the pisiform; however, hypothenar pain persisted when he flexed his right wrist. Furthermore, his grip weakness did not improve.

**Figure 2:**
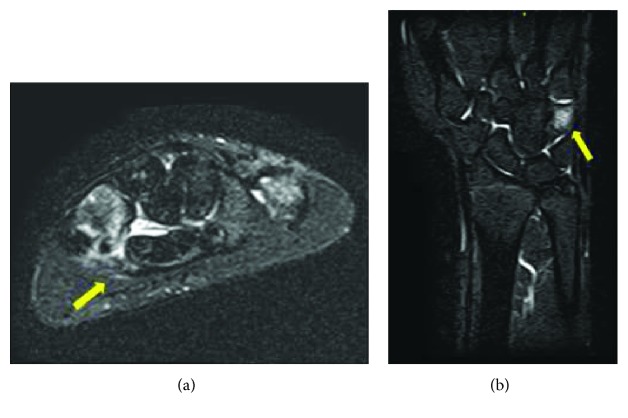
Magnetic resonance imaging (MRI) images. (a) Axial T2-weighted STIR image. (b) Coronal T2-weighted STIR image. Intensity changes around the hamate hook suggest inflammation (indicated by arrows).

**Figure 3:**
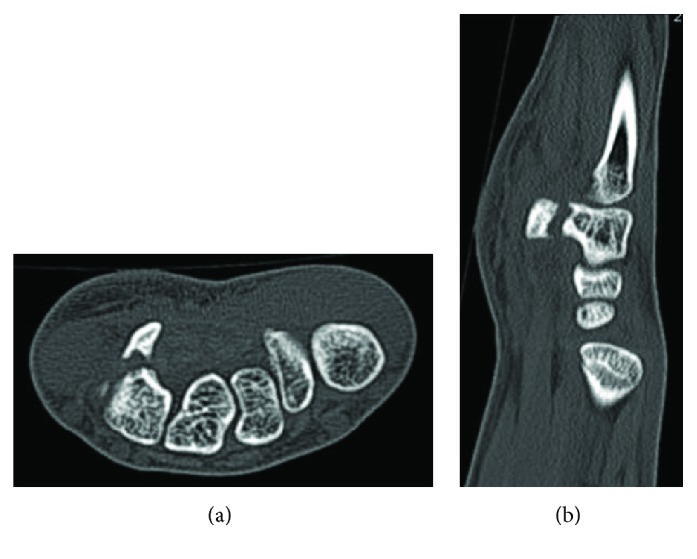
Computed tomography (CT) images. (a) Axial CT image. (b) Sagittal CT image.

To diagnose the injury, magnetic resonance imaging (MRI; T1-weighted, T2-weighted, T2-weighted STIR) of the affected region was performed. The right wrist showed intensity changes around the hamate hook, suggesting inflammation (Figure 2). A computed tomography scan finally showed fracture of the hamate hook (Figure 3). Upon a careful reexamination, tenderness was observed on the hamate hook.

**Figure 4:**
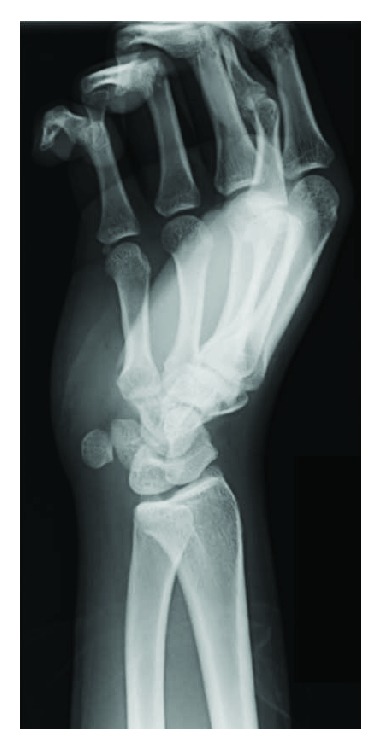
Postoperative radiograph in supinated oblique position.

Three weeks after his initial presentation, we surgically removed the hamate hook fragment. Mild synovitis of the flexor tendon of the small finger was intraoperatively found but not at the site of injury itself. Radiograph taken after the surgery showed that the hamate hook fragment was completely removed (Figure 4).

He was advised not to play water polo for 3 weeks after the surgery, and he did not resume practice until 4 weeks. Three months after the surgery, mild tenderness around the surgical scar was reported; however, his grip strength of the right hand improved and was measured as 44 kg. One year after the surgery, he was completely pain-free, and the grip strength of his right hand had further improved to 46 kg. He achieved the full range of motion in the wrist (95° extension, 115° flexion) and felt no pain around his right wrist at any time, including while playing water polo. The most recent Disabilities of the Arm, Shoulder and Hand score was 2.6 points.

## 3. Discussion

Fractures of the hamate account for 2% of all carpal fractures. The most common causes are racket sports, baseball, and golf, and the main mechanism of fracture is the direct injury to the hamate hook [1].

Several reports have indicated fracture of the hamate hook without direct trauma to the hamate hook itself, caused by some other indirect mechanism. How Kit et al. [2] have reported a case of a 23-year-old professional bowler who had gradually increasing hypothenar pain after he changed his bowling technique and ball-drilling layout. He was diagnosed with stress fracture of the hamate hook without previous direct trauma to the wrist. Bowler's wrists were hyperextended, pronated, and deviated to spin the ball because of its weight during the release. It was also noted that changes in the bowling technique and ball-drilling layout produced indirect forces on the hamate hook via the attached ligaments and strain on the flexor tendons. Lutter et al. [3] have reported fractures of the hamate hook in 10 of 12 high-level rock climbers presenting with ulnar wrist pain without any history of direct trauma. Rock climbing does not usually cause direct trauma to the hamate hook; however, it was noted that the side-undercling position that is repetitively used by rock climbers forces the wrists into ulnar deviation and strains the flexor tendon and, thus, may serve as an indirect mechanism of the fracture by compressing the hamate hook. Scheufler et al. [4] have also reported a high incidence of fractures of the hamate hook in underwater rugby players. The flexor digiti minimi brevis and abductor digiti minimi attach to the hamate hook, which works as a pulley for the flexor tendons of the ring and small fingers when the wrist undergoes ulnar deviation or during strong gripping [4, 5]. Because the ball is firmly held with an outstretched hand, strong indirect forces are produced by the muscles and ligaments attached to the hamate hook, as well as the flexor tendons of the ring and small fingers, leading to a high incidence of fractures of the hamate hook in underwater rugby players.

In our case, the fracture of the hamate hook in the water polo player was difficult to detect and diagnose because he had no previous typical direct trauma to his right wrist. However, because the onset of his wrist pain was nonspecific and similar to previous reports of indirect mechanisms, we considered that this may be a case of stress fracture of the hamate hook. In such cases, MRI is useful in diagnosing the fracture.

The following two indirect mechanisms led to stress fracture of the hamate hook in the water polo player. The first mechanism was the indirect forces that resulted from the strong ulnar deviation of the wrist during ball throwing. In water polo, putting snap on the ball is needed to spin the ball while throwing it. Particularly, pushing and knuckling are unique motions used to throw a ball in water polo, which cause strong ulnar deviations of the wrist at ball release, similar to the side-undercling position of rock climbers [3]. Extreme deviation of the wrist likely puts strain on the flexor tendons producing strong forces on the hamate hook. The second mechanism was the indirect forces due to strongly holding the ball with one hand. The ball used in underwater rugby has a circumference of 52–54 cm for males and 49–51 cm for females and adolescents [4], whereas that used in water polo has a much larger circumference of 68–71 cm for males and 65–67 cm for females. Furthermore, water polo players hold the larger ball with one hand in the same manner as underwater rugby players. Thus, it is likely that the indirect forces acting on the hamate hook via the muscles, ligaments, and flexor tendons are greater in water polo players than in underwater rugby players because of the larger ball size. Based on these two mechanisms, the fracture in our case was considered stress fracture of the hamate hook and occurred because of a motion and position of the wrist unique to water polo.

No previous studies relating to stress fractures of the hamate hook in water polo players have been reported. Our patient's range of motion in the bilateral wrists at 1 year after the surgery was wider than normal but cannot be regarded as so-called general joint laxity and may lead to a greater strain on the flexor tendons.
